# Prognostic Factors and Treatment Effect Modifiers for Physical Health, Opioid Prescription, and Health Care Utilization in Patients With Musculoskeletal Disorders in Primary Care: Exploratory Secondary Analysis of the STEMS Randomized Trial of Direct Access to Physical Therapist–Led Care

**DOI:** 10.1093/ptj/pzae066

**Published:** 2024-05-02

**Authors:** James Zouch, Nazim Bhimani, André Bussières, Manuela L Ferreira, Nadine E Foster, Paulo Ferreira

**Affiliations:** Faculty of Medicine and Health, The University of Sydney, Sydney, New South Wales, Australia; Faculty of Medicine and Health, The University of Sydney, Sydney, New South Wales, Australia; Upper Gastrointestinal Surgical Unit, Royal North Shore Hospital, Sydney, New South Wales, Australia; Département de Chiropratique, Université du Québec à Trois-Rivières, Trois-Rivières, Quebec, Canada; School of Physical & Occupational Therapy, McGill University, Montreal, Quebec, Canada; Sydney Musculoskeletal Health, The Kolling Institute, Faculty of Medicine and Health, The University of Sydney, Sydney, New South Wales, Australia; Surgical Treatment and Rehabilitation Service (STARS) Education and Research Alliance, The University of Queensland and Metro North Hospital and Health Service, Herston, Queensland, Australia; Primary Care Centre Versus Arthritis, School of Medicine, Keele University, Newcastle-under-Lyme, Staffordshire, United Kingdom; Faculty of Medicine and Health, Musculoskeletal Pain Hub, Charles Perkins Centre, Sydney Musculoskeletal Health, The University of Sydney, Sydney, New South Wales, Australia

**Keywords:** Back Pain, Effect Modifiers, Musculoskeletal Pain, Primary Health Care, Prognosis

## Abstract

**Objective:**

The aims of the study were to identify prognostic factors associated with health care outcomes in patients with musculoskeletal (MSK) conditions in primary care and to determine whether characteristics associated with choice of care modify treatment effects of a direct-access physical therapist–led pathway in addition to general practitioner (GP)–led care compared to GP-led care alone.

**Methods:**

A secondary analysis of a 2-parallel-arm, cluster randomized controlled trial involving general practices in the United Kingdom was conducted. Practices were randomized to continue offering GP-led care or to also offer a direct-access physical therapist–led pathway. Data from adults with MSK conditions who completed the 6-month follow-up questionnaire were analyzed. Outcomes included physical health, opioid prescription, and self-reported health care utilization over 6 months. Treatment effect modifiers were selected a priori from associations in observational studies. Multivariable regression models identified potential prognostic factors, and interaction analysis tested for potential treatment effect modifiers.

**Results:**

Analysis of 767 participants indicated that baseline pain self-efficacy, pain severity, and having low back pain statistically predicted outcomes at 6 months. Higher pain self-efficacy scores at baseline were associated with improved physical health scores, reduced opioid prescription, and less health care utilization. Higher bodily pain at baseline and having low back pain were associated with worse physical health scores and increased opioid prescription. Main interaction analyses did not reveal that patients’ age, level of education, duration of symptoms, or MSK presentation influenced response to treatment, but visual trends suggested those in the older age group proceeded to fewer opioid prescriptions and utilized less health care when offered direct access to physical therapy.

**Conclusions:**

Patients with MSK conditions with lower levels of pain self-efficacy, higher pain severity, and presenting with low back pain have less favorable clinical and health care outcomes in primary care. Prespecified characteristics did not modify the treatment effect of the offer of a direct-access physical therapist–led pathway compared to GP-led care.

**Impact:**

Patients with MSK conditions receiving primary care in the form of direct-access physical therapist–led or GP-led care who have lower levels of self-efficacy, higher pain severity, and low back pain are likely to have a less favorable prognosis. Age and duration of symptoms should be explored as potential patient characteristics that modify the treatment response to a direct-access physical therapist–led model of care.

## Introduction

Musculoskeletal (MSK) conditions, including low back pain and osteoarthritis, are a growing global burden, ^1^ increasing the strain on primary health care systems worldwide.^2^^,^^3^ General practitioners (GPs), also referred to as primary care physicians, who traditionally provide first-contact care for patients entering the health care system, are tasked with managing an increasing number of patients with MSK conditions. The increased burden is leading to increased patient waiting times,^4^ physician workloads, and health care costs.^5^ Reports from primary health care systems in Australia,^6^^,^^7^ the United States of America,^8^ and the United Kingdom,^9^ indicate workload may already be unsustainable and requires urgent consideration of new models of primary care and expanded primary care teams.^10^

One promising model of care that could decrease the MSK condition workload on GPs is to offer direct access to physical therapist–led primary care to patients with MSK conditions. This model provides patients with access to an established workforce of clinicians trained in the diagnosis and management of MSK conditions without need for an initial GP consultation.^11^ Emerging evidence suggests direct access to physical therapist–led care is safe, as effective as GP-led care, and may reduce health care utilization and cost of care for patients with MSK conditions.^12^ For patients with low back pain, starting care with a physical therapist may reduce the odds of being prescribed opioids^13^ compared to accessing traditional GP-led care.^14–17^ Although preliminary research is promising, a greater understanding of factors associated with success in pragmatic direct-access physical therapist–led models of care are needed to identify characteristics of patients who are most likely to benefit.

Factors influencing a patient’s choice of care provider, care seeking, and access to primary care must be considered. Characteristics such as younger age,^18^^,^^19^ higher levels of education,^18–21^ and shorter duration of symptoms are more common in patients choosing to directly access physical therapist–led primary care than in those initiating care with a GP.^18^ These characteristics may reflect the nature of funding of health care systems, and the disparity in resources of patients. They are also known to be associated with better MSK clinical outcomes such as physical disability, quality of life,^22^ and symptom acceptability.^23^ Thus, exploring possible differences in treatment effects within these patient subgroups may provide important information about the characteristics that influence response to treatment in pragmatic direct access to physical therapist–led pathways compared to usual GP-led care.

Although considerable research has focused on prognostic factors associated with clinical outcomes such as physical health/function, pain and quality of life in patients with MSK conditions,^24^ less is known about factors associated with opioid prescription^25^ and health care utilization.^26^ These outcomes are important when considering the limited health care resources, increasing demand in primary care and the proposed benefits of direct access to a physical therapist–led model of care. Opioid prescription and health care utilization (ie, investigations and procedures beyond the health care consultation^27^) often account for the largest portion of health care costs and can represent an unnecessary escalation of care in the absence of more serious MSK pathology.^27^ Additionally, models of direct-access physical therapy do not appear to be superior to usual GP-led care^12^ in terms of clinical outcomes, placing a greater impetus on understanding the impact of direct-access physical therapy on outcomes such as health care utilization and opioid prescription.

Using data from a randomized controlled trial (RCT) of direct access to physical therapy for adults with MSK conditions (the UK STEMS pilot trial^28^), the 2 aims of the current study were to identify characteristics of patients presenting to UK primary care that are prognostic of health care utilization, opioid prescription, and physical health over 6 months and to explore whether previously identified patient characteristics associated with either the choice of first-contact care or variable treatment outcomes modify the treatment effects between usual GP-led care or GP-led care with the additional offer of direct access to physical therapist–led care.

## Methods

We conducted a secondary analysis of data collected from the STEMS trial (registration ISRCTN23378642). STEMS was a large pilot 2-parallel-arm, cluster RCT involving 4 general practices in the United Kingdom.^28^ General practices were randomized to continue offering usual GP-led care (control arm) or GP-led care with the additional offer for patients to directly access physical therapist–led care (intervention arm). As practices, and not individual participants, were the unit of randomization, in the intervention arm patients could see a GP, opt to self-refer to a physical therapist for their MSK condition, or they could be directed to do so after an interaction with another staff member at the practice. Patient direct access to physical therapist services was not available in the control arm. In the intervention arm 36% of participants accessed physical therapy (33% directly), meaning the majority of patients received GP-led care. The STEMS trial offers the ability to analyze the effects of the offer of direct access rather than a comparison of only GP-led care versus only physical therapist–led care.

Patients were eligible if they were > 18 years old and were consulting about an MSK condition and ineligible if they were undergoing palliative care, had severe learning disabilities, were housebound or living in a nursing home, were unable to communicate in English, or were unable to provide consent to participate in data collection. Participants were recruited from June 2013 to January 2014. Eligible patients completed self-reported questionnaires (via mailed forms) at baseline and at 2, 6, and 12 months. General practice and health care records were accessed, with consent, for medication prescription data.^28^ The primary aim of the STEMS pilot trial was to assess the feasibility of a future large trial, using an anticipated primary clinical outcome of physical health (Short-Form Health Survey, version 2 [SF-36v2]) and cost-effectiveness analysis, to compare the addition of direct-access physical therapy to usual GP-led care with usual GP-led care alone. Original exploratory and descriptive analyses did not indicate a clinically meaningful difference (>2 points) between the treatment arms for physical health at any time point. The National Health Service costs per patients were slightly lower (£940) in the intervention arm than in the control arm (£951) but with large uncertainty in the estimates (95% CI of difference = −363.27 to 340.83).^28^

### Prognostic Factors

Baseline variables collected through the STEMS questionnaires were analyzed in multivariable regression modeling to explore prognostic factors associated with outcomes, after adjusting for the intervention received. Because of the exploratory nature of this secondary analysis, all baseline variables (Tab. 1) with plausible prognostic characteristics^24^ were included in base models^29^ (Suppl. Tab. 1).

**Table 1:** Baseline Characteristics of Participants in the STEMS Trial With Completed Follow-up at 6 Months*^a^*

**Characteristic**	**Sample Receiving Direct Access (*n* = 326)**	**Sample Receiving Usual Care (*n* = 441)**	**Total Sample (*N* = 767)**
Nonclinical			
Age, mean (SD)	57.2 (14.2)	59.6 (14)	58.3 (14.1)
Sex, female	199 (61.0)	252 (57.1)	451 (58.8)
Ethnicity: white	321 (98.5)	424 (96.1)	745 (97.1)
Employment in paid work	110 (33.7)	136 (30.8)	246 (32.1)
Education level			
High	30 (9.2)	56 (12.7)	86 (11.2)
Intermediate	99 (30.4)	116 (26.3)	215 (28.0)
No qualification/low	156 (47.9)	208 (47.2)	364 (47.5)
Deprivation tertile			
Most deprived third	75 (23.0)	122 (27.7)	197 (25.7)
Middle third	110 (33.7)	143 (32.4)	253 (33.0)
Least deprived third	141 (43.3)	176 (39.9)	317 (41.3)
Health literacy: health information difficult to understand	180 (55.2)	242 (54.9)	422 (55.0)
Clinical			
Physical health, determined with SF-36v2 PCS subscale at baseline, mean (SD)	36.8 (9.8)	35.8 (10.1)	36.3 (10.0)
Mental health, determined with SF-36v2 MCS subscale, median (IQR)	43.8 (31.9–55.3)	48 (35.6–56.6)	44.4 (33.9–56.3)
Bodily pain in past week: severe/very severe	147 (45.1)	190 (43.1)	337 (43.9)
Episode duration:			
Pain for <6 weeks	67 (20.6)	113 (25.6)	180 (23.5)
Pain for 6–12 weeks	38 (11.7)	145 (10.2)	83 (10.8)
Pain for >12 weeks	203 (62.3)	259 (58.7)	462 (60.3)
Widespread pain: yes*^b^*	109 (33.4)	165 (37.4)	274 (35.7)
Comorbidities: ≥2 other conditions	189 (58.0)	266 (60.3)	455 (59.3)
Pain self-efficacy, determined with PSEQ, mean (SD)	33.15 (16.4)	34.4 (16.2)	33.9 (16.3)
MSK pain presentation: low back pain	179 (54.9)	242 (54.9)	422 (55.0)

^a^ Data are reported as numbers (percentages) of participants unless otherwise indicated. Percentages may not sum to 100 because of missing data in some variables. IQR = interquartile range; MCS = mental component summary; MSK = musculoskeletal; PCS = physical component summary; PSEQ = Pain Self-Efficacy Questionnaire; SF-36v2 = Short-Form Health Survey, version 2.

*^b^* Based on the American College of Rheumatology definition.^30^

### Treatment Effect Modifiers

Patient characteristics associated with choice of access to care (ie, age, duration of symptoms, and level of education) as well as patient characteristics associated with improved outcomes (ie, patients with low back pain) when choosing direct-access physical therapy compared to GP-led care were identified a priori from observational studies and a recent systematic review on nonmedical triage for MSK pain.^12^^,^^18–21^^,^^31^ These characteristics have been associated with variability of clinical outcomes in MSK conditions and were included as potential treatment effect modifiers for this secondary analysis. Therefore, the following variables were analyzed as potential treatment effect modifiers: age (≤44, 45–64, or ≥ 65 years),^18^^,^^19^^,^^32^ pain duration (<6 weeks, 6–12 weeks, or > 12 weeks),^33^ level of education (high [degree or postgraduate qualification], intermediate [A levels, completion of high school, preuniversity required courses, or work-related qualification], and low [General Certificate of Secondary Education, American high school diploma equivalent, or no qualification]), and self-reported MSK pain location (low back pain vs other MSK pain presentations).^34^

### Outcomes

Outcomes previously suggested to improve following direct-access physical therapist–led care^12^^,^^13^ (opioid prescription and health care utilization) were considered outcomes in the prognostic analyses and treatment modification (interaction) analyses. The key clinical outcome of the STEMS trial (physical health) was considered an outcome in the prognostic analyses only, to enable comparison of identified prognostic factors with the current evidence base.

The outcome physical health was analyzed at 6 months (the time point with the highest response rate from the STEMS trial), and the outcomes opioid prescription and health care utilization were analyzed from baseline to the 6-month follow-up.

For opioid prescription, medication prescribed by participating GPs and obtained from practice medical records (collected in the STEMS trial) from the date of participant consent for data collection was recoded as opioid-based medication (eg, codeine, co-codamol, or tramadol) and recorded as a binary outcome (“yes” or “no”).

For health care utilization, a total count of investigations or procedures was coded per participant, using responses from a self-reported questionnaire mailed to participants 6 months from the indexed consultation with the following question: In the last 6 months have you had any investigations or treatments (eg, x-ray, MRI [magnetic resonance imaging], surgery, injection) in the NHS (National Health Service) or privately for your pain or pain related symptoms? Participants then recorded the type of treatment or investigation they received and the reason (eg, knee pain), alongside the number of treatments/investigations. All responses could be coded under the following categories: magnetic resonance imaging, x-ray, computerized tomography scan, ultrasound scan, injection, or surgery. Treatments/investigations that were not related to MSK conditions (eg, “echocardiogram for heart issues”) were excluded.

Physical health was assessed using the physical component summary (PCS) subscale of the SF-36v2, a generic measure of health, assessing 8 domains: physical functioning, bodily pain, limitations due to physical health problems, limitations due to personal or emotional problems, emotional well-being, social functioning, energy/fatigue, and general health perceptions.^35^ A higher score was representative of better physical health. The SF-36v2 PCS was the primary outcome in the STEMS trial.

### Analysis

Baseline characteristics of participants who also completed questionnaire at 6 months were analyzed descriptively using means and standard deviations, or medians and interquartile ranges (continuous variables), and proportions (categorical variables). Where appropriate,^36–39^ multiple imputation was performed on the basis of a missing at random assumption using a multiple imputation by chained equations approach^37^ (see Suppl. Material for multiple imputation process).

### Model Building

First, we created separate regression models to explore prognostic characteristics associated with each of the 3 outcomes, irrespective of intervention arm allocation: linear regression for physical health (SF-36v2 PCS), logistic regression for opioid prescription, and Poisson regression for health care utilization. A variance inflation factor was computed for all variables used in the model and potential multicollinearity was identified by a variance inflation factor of >10. Models were created for multiple imputed and complete case datasets. A backward stepwise progression approach was used to identify characteristics predictive of outcome with a cutoff set at *P* > .05. This was conducted by removing 1 variable at a time and assessing the impact on remaining regression coefficients in the model. Model variability was estimated using the “mibeta” command [authored by Yulia Marchenko] to estimate adjusted *R*^2^ values for multiple imputed linear regression models and the concordance statistic (c) for logistic models. Results for physical health (SF-36v2 PCS) were presented as regression coefficients (b), for opioid prescription as adjusted odds ratio (aOR), and for health care utilization, as an incidence rate ratio (IRR). Results were reported for multiple imputed datasets with complete case analysis presented for comparison purposes. All values were reported with 95% CIs.

Second, we explored treatment effect modification using the interaction between trial arms, and patient characteristic identified a priori. Analysis comparing the trial arms ensured adequate subgroup sample sizes and reflected the pragmatic nature of the trial, rather than selectively subgrouping only those participants who directly accessed physical therapist–led care. We constructed separate models using logistic regression for opioid prescription and Poisson regression for health care utilization, with interaction variables fitted in the base model. Postestimation commands served to determine significance of the main interaction effect. Potential treatment effect modifiers were evaluated using interaction analysis and reported descriptively using interaction plots with pairwise comparisons estimated using marginal means and 95% CIs.^40^

All analyses were performed using Stata version 17 software package (StataCorp LLC, College Station, TX, USA). The University of Sydney exempted this secondary analysis of existing RCT data from ethical approval as the STEMS trial dataset was available as grouped, nonidentifiable data.

## Results

Of the 978 participants included in the STEMS trial, 767 completed 6-month questionnaires (Suppl. Figure). Baseline characteristics (potential prognostic factors) of those who completed 6-month follow-up were similar between the 2 trial arms (Tab. 1). From those, 457 (59.6%) had complete observations for all variables used in this secondary analysis. Baseline data were missing for participants’ level of education (13.3%), Pain Self-Efficacy Questionnaire (8.9%), and pain duration (5.5%). Follow-up data were missing for paid employment (22.9%) and health care utilization (16.6%). See the Supplemental Material for missing data summary (Suppl. Tab. 6–8) and variance inflation factor results. Counts of health care utilization were similar between treatment arms at 6 months with majority of participants having reported no health care utilization, and a similar proportion of patients having utilized 2 or more health care procedures in each arm (Suppl. Tab. 2). For information separated by individual investigations and procedures, see Supplementary Table 3. Opioid prescription rates were also similar between treatment groups (Suppl. Tab. 4).

**Table 2:** Multivariable Logistic Regression Model Showing Baseline Prognostics Factors Associated With Opioid Prescription Over 6 Months*^a^*

**Variable**	**Multiple Imputation**		**Complete Case Analyses**	
	**Adjusted OR (95% CI)**	***P***	**Adjusted OR (95% CI)**	***P***
Pain self-efficacy, determined with PSEQ	0.96 (0.95–0.98)	<.001	0.96 (0.94–0.98)	<.001
Mental health, determined with SF-36v2 MCS subscale	N/A	N/A	0.98 (0.958–0.997)	.022
MSK pain presentation				
All (reference)				
Lower back	1.56 (1.10–2.21)	.013	N/A	N/A
Level of education		.001		.032
Low/no qualification (reference)				
Intermediate	0.80 (0.56–1.16)		0.83 (0.51–1.33)	
High	0.35 (0.20–0.61)		0.37 (0.18–0.78)	
Bodily pain		<.001		.017
No/mild (reference)				
Moderate	1.52 (0.92–2.52)		1.52 (0.75–3.07)	
Severe–very severe	3.29 (1.88–5.78)		2.81 (1.28–6.16)	
Sex				
Male (reference)				
Female	N/A		0.51 (0.32–0.82)	.005
Concordance statistic	0.78		0.81	

^a^ After controlling for intervention arm. MCS = mental component summary; MSK = musculoskeletal; N/A = not applicable; OR = odds ratio; PSEQ = Pain Self-Efficacy Questionnaire; SF-36v2 = Short-Form Health Survey, version 2.

### Exploratory Prognostic Factors

#### Opioid Prescription

Baseline variables identified as prognostic factors for opioid prescription, from baseline to 6-months, included pain self-efficacy, MSK pain presentation, level of education and bodily pain at baseline (Tab. 2). Those with higher pain self-efficacy (aOR = 0.96; 95% CI = 0.95 to 0.98), as well as those with higher level of education (aOR = 0.35; 95% CI = 0.20 to 0.61) compared to those with the lowest levels of or no formal education, were less likely to be prescribed opioids. Those with higher pain severity (aOR = 3.29; 95% CI = 1.88 to 5.78), compared to those with no or mild pain, and those with low back pain (aOR = 1.56; 95% CI = 1.10 to 2.21), compared to those with other MSK pain presentations were more likely to be prescribed an opioid.

#### Health Care Utilization

Baseline variables identified as prognostic factors for health care utilization, from baseline to 6-months, included pain self-efficacy and bodily pain (Tab. 3). Higher pain self-efficacy was associated with a reduction in health care utilization (IRR = 0.99; 95% CI = 0.981 to 0.998), and bodily pain severity was associated with increased health care utilization; those with severe to very severe pain at baseline had a rate of health care utilization 1.82 times greater than those with no or mild pain (IRR = 1.82; 95% CI = 1.15 to 2.86). The association between those presenting with low back pain and health care utilization rate was 1.26 times higher than those with any other MSK pain presentation, but this relationship could vary in magnitude and direction in broad populations on the basis of the CI around our estimate (IRR = 1.26; 95% CI = 0.98 to 1.60).

**Table 3:** Multivariable Poisson Regression Showing Baseline Prognostic Factors Associated With Health Care Utilization Over 6 Months*^a^*

**Variable**	**Multiple Imputation**		**Complete Case Analyses**	
	**IRR (95% CI)**	***P***	**IRR (95% CI)**	***P***
Pain self-efficacy, as determined with PSEQ	0.99 (0.981–0.998)	.016	0.98 (0.974–0.989)	<.001
Bodily pain		.020	N/A	N/A
No/mild (reference)				
Moderate	1.42 (0.94–2.15)			
Severe–very severe	1.82 (1.15–2.86)			
MSK pain presentation			N/A	N/A
All (reference)				
Lower back	1.26 (0.98–1.60)	.066*^b^*		
Pseudo *R*^2^	N/A		0.03	

^a^ After controlling for intervention arm. IRR = incidence rate ratio; MSK = musculoskeletal; N/A = not applicable; PSEQ = Pain Self-Efficacy Questionnaire.

*^b^* Not statistically significant.

#### Physical Health

Baseline variables identified as prognostic factors for physical health at 6 months included age, pain duration, bodily pain, general health, MSK pain presentation, comorbidities, widespread pain, pain self-efficacy and mental health (Suppl. Tab. 5). Only baseline pain self-efficacy was positively associated with physical health (b = 0.23; 95% CI = 0.17 to 0.29). Older age (b = −3.59; 95% CI = −25.38 to −1.80), pain symptoms greater than 12 weeks duration (b = −3.16; 95% CI = −4.61 to −1.71), higher severity of bodily pain (b = −4.49; 95% CI = −6.54 to −2.44), poor general health (b = −11.40; 95% CI = −14.74 to −8.07), presence of 2 or more comorbidities (b = −2.30; 95% CI = −3.62 to −0.99), presence of widespread pain (b = −1.60; 95% CI = −3.07 to −0.13), lower mental health scores (b = −0.07; 95% CI = −0.13 to −0.01), and having low back pain (b = −1.62; 95% CI = −3.05 to −0.19) were all prognostic of worse physical health at 6 months.

### Treatment Effect Modifiers

#### Opioid Prescription

**Figure 1:**
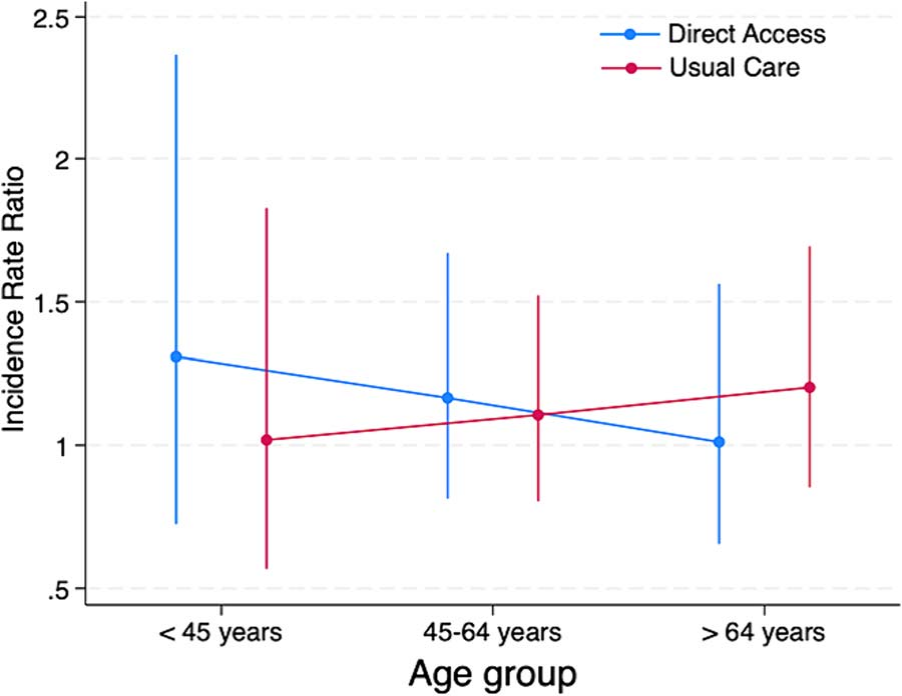
Model estimated margins for age effect modification of treatment for opioid prescription outcome.

A treatment effect modifier was not observed for age (*F*_2,2.4e+06_ = 0.4; *P* = .672), pain duration (*F*_1,14,057_ = 0.12; *P* = .883), level of education (*F*_2,6286_ = 0.18; *P* = .833), or MSK pain presentation (*F*_1,1.4e+06_ = 0.96; *P* = .326). Visual trends observed in interaction plots suggested the older age subgroup (those >64 years of age) may have responded better to the intervention offering direct access to physical therapist–led care, with large uncertainty in the precision of these estimates (Fig. 1).

#### Health Care Utilization

**Figure 2:**
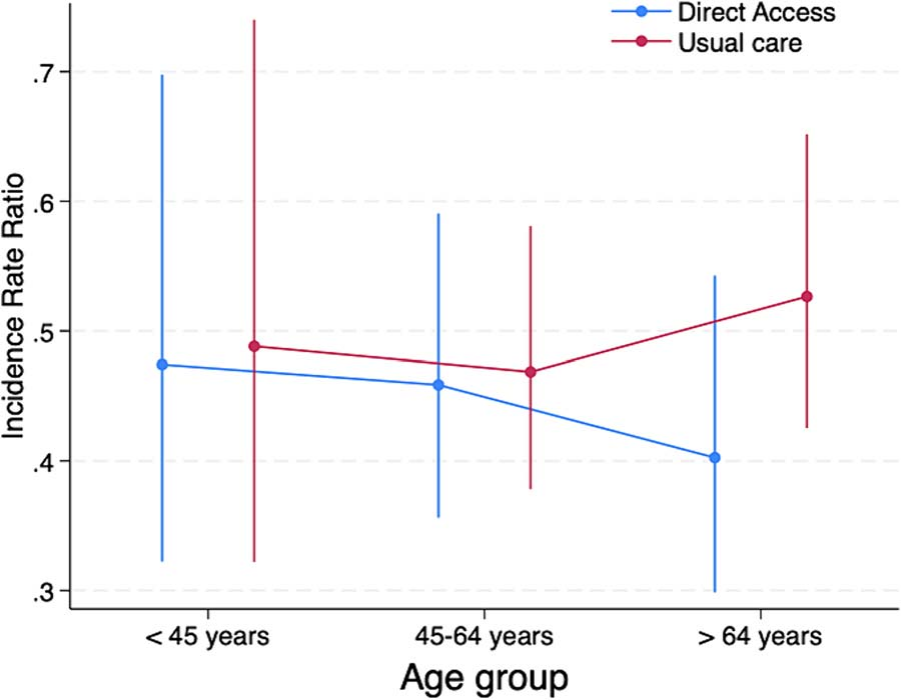
Model estimated margins for age effect modification of treatment for health care utilization outcome.

**Figure 3:**
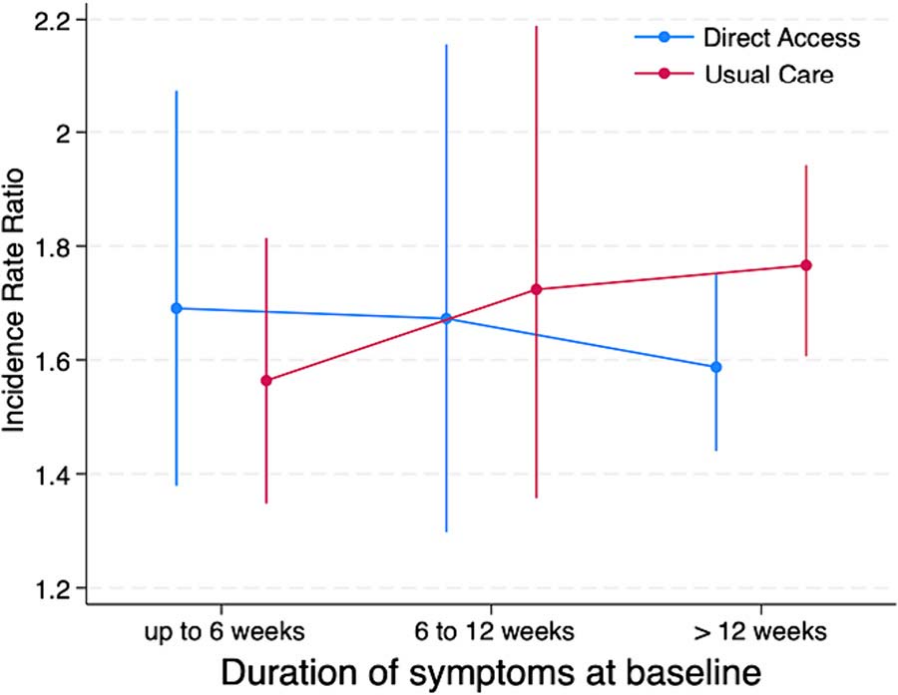
Model estimated margins for pain duration effect modification of treatment for health care utilization outcome.

A treatment effect modifier effect was not observed for age (*F*_2,1877_ = 0.51; *P* = .603), pain duration (*F*_2,2386_ = 0.88; *P* = .417), level of education (*F*_2,2193_ = 0.46; *P* = .631), or MSK pain presentation (*F*_1,2061_ = 0.04; *P* = .842). Visual trends observed in interaction plots suggested that the older subgroup (>64 years old) and those with a longer duration of symptoms (>12 weeks) may have responded better to the intervention offering direct access to physical therapist–led care, with large uncertainty in the precision of these estimates (Fig. 2 and Fig. 3).

## Discussion

Results from this exploratory secondary analysis showed that in terms of prognostic factors, patients with higher pain self-efficacy had better physical health, reduced odds of being prescribed opioids, and utilized less health care, 6 months after initiating care. In contrast, patients with higher bodily pain at baseline had worse physical health, had increased odds of being prescribed opioids, and utilized more health care 6 months after initiating care compared to those with no or mild bodily pain at baseline. Those with low back pain had worse physical health and increased odds of being prescribed opioids over 6 months from initiating care compared to those with any other MSK pain presentation. Additionally, modeling from this study suggests that prognostic factors may be outcome-specific; for example, prognostic variables associated with opioid prescription outcome might differ from those associated with physical health outcome. Our exploratory treatment effect modifier analysis did not find evidence that patients’ age, level of education, duration of symptoms, or MSK pain presentation (low back pain) appeared to influence response to treatment, where the treatment involved a model of care where patients were also offered a direct-access pathway to physical therapy compared to usual GP-led care alone.

### Prognostic Factors

The prognostic factors associated with physical health identified in this study (ie, pain duration, bodily pain severity, general health, comorbidities, widespread pain, mental health and pain self-efficacy) are consistent with recent systematic and umbrella review findings on prognostic factors for patient reported clinical outcomes such as physical function in MSK pain.^24^^,^^41^ Identification of generic prognostic factors (ie, pain self-efficacy, baseline pain severity, and MSK pain presentation) associated with the outcomes of opioid prescription and health care utilization, are an interesting finding. Though generalizable to this sample only, it is useful to interpret the difference in magnitude of effect that these variables had across outcomes. For example, a participant presenting with a 10-point higher score on the pain self-efficacy scale (indicating a higher pain self-efficacy compared to another participant) was associated with a 10% lower rate of health care utilization over 6 months. A 10-point higher pain self-efficacy score had a greater effect on opioid prescription, with a 44% reduction in the odds of receiving an opioid prescription. Comparison with other cohorts with MSK conditions provides context to the consistency of these associations and potential clinical relevance. Limited previous research attempting to identify prognostic factors for these outcomes in populations with MSK conditions has also observed that pain self-efficacy (posttreatment) is a strong independent predictor of medication use at 1 year follow-up in a cohort of worker’s compensation claimants.^42^ Pain severity, as a multivariate predictor of both patient reported opioid prescription in a military cohort^43^ and opioid use, following an episode of physical therapy in a large cohort of patients with MSK pain.^27^ Within the same study, and contrary to our findings, pain severity across a range of services (eg, injection, surgery, imaging) was not shown to be predictive of health care utilization. Together, these findings suggest that patients with MSK pain, higher pain self-efficacy, and lower pain severity may be less likely to request/be prescribed opioid medication, potentially reflecting their confidence in their ability to complete their everyday tasks despite pain^44^; however, the association with health care utilization is unclear.

Previous research has shown that low back pain presentations compared to other MSK complaints, are associated with greater odds of emergency department visits,^27^ and opioid prescription,^45^^,^^46^ providing additional evidence that MSK pain presentation may be an important prognostic factor to examine in the MSK field. The association between low back pain and these outcomes may reflect a divergent management approach associated with specific MSK pain sites/conditions.^47^ For example, patients with low back pain may be more routinely referred for imaging by practitioners in primary care to meet patients’ needs or because of concerns about potential malpractice claims.^48^ The nature of these associations is beyond the scope of this analysis, and validation research is needed to determine the importance of these findings.

In the current study, we found multivariate prognostic factors associated with physical health outcome, differed to those associated with opioid prescription (pain self-efficacy, mental health, MSK pain presentation, level of education, bodily pain) and health care utilization outcomes (pain self-efficacy, bodily pain). Emerging evidence suggests prognostic factors are distinct for health care utilization and opioid prescription outcomes^27^^,^^43^ and may not reflect prognostic factors typically associated with outcomes such as physical health or function. Although accuracy of prognostic models was not the primary focus of this study, prognostic characteristics associated with opioid prescription over 6 months showed good discriminatory ability (concordance statistic = 0.78) to predict opioid prescription outcome in this sample, which differed significantly from the prognostic models associated with physical health and health care utilization. The observation that prognostic factors are outcome specific is supported by a recent systematic review investigating prognostic factors following an initial opioid prescription for acute MSK injuries.^25^ Though pooling of data was limited by the small number of included studies, variables such as education, age, substance use disorder, history of self-injury and receipt of Medicaid were consistently associated with prolonged opioid use in this review, and differ to those typically associated with clinical outcomes of physical health or function.^24^ Similarly, recent predictive modeling research in cohorts with MSK conditions^27^^,^^43^ suggests that factors that predict health care utilization are highly specific—for example, that predictors of emergency room visits for MSK care are distinct from predictors of diagnostic imaging. Comparison with other literature is limited by the mixed definition of the term “health care utilization,“^49^ the combination of multiple services (eg, opioid prescription, consultations, imaging, injections) into 1 outcome measure,^49–51^ and limited research in cohorts with MSK conditions.^27^^,^^43^

Although opioid prescription and health care utilization outcomes are not traditionally used as primary outcome measures with which to determine the effectiveness of MSK health care, they are becoming increasingly important in establishing the effectiveness of new models of care for MSK conditions.^12^ Future research is needed to identify characteristics prognostic of these outcomes in cohorts with MSK conditions, using uniform definitions and distinct, nongrouped outcomes which may provide a greater understanding of what factors, and ultimately, which treatment models/pathways are most effective at reducing unnecessary health care utilization.

### Treatment Modifiers

We did not find robust evidence that patients with MSK conditions and certain characteristics identified a priori (age, pain duration, level of education, or MSK pain presentation of low back pain), respond differently to the offer of direct-access physical therapy compared to GP-led care alone. Although no significant main interaction effects were observed, and the exploratory nature of pairwise comparison of subgroups precludes over-interpretation of findings,^40^ trends observed in interaction plots may inform future research. Visual trends suggest participants in the older age group (those >64 years old) and those with a longer duration of pain at baseline (>12 weeks) were less likely to utilize further health care, with those in the older age group also less likely to be prescribed opioids, when they were offered direct-access physical therapy compared to usual GP-led care alone. Confirming these trends in future large pragmatic trials is important considering consistent evidence that indicates that some subgroups of patients with MSK conditions, such as those of older age, have higher rates of long-term opioid prescription within UK,^52^ Australian,^53^ and Dutch^54^ primary care systems. Similarly, older age groups appear to utilization health care at higher rates.^55^ Older patients with MSK conditions often require specific consideration by clinicians^52^ given their propensity for multimorbidities,^56^ polypharmacy,^57^ persistent pain^58^, and increased risk of medication side effects.^59^ A nonpharmacological first-line approach, such as direct access to physical therapy in primary care, may provide a solution to reduce long-term opioid prescription in this age group. This argument is supported by observational studies which have demonstrated an association between patients who consult a physical therapist early in their episode of care and reduced odds of opioid prescription in knee, neck, shoulder, and lower back cohorts.^60^ Although direct-access physical therapy models of care offer a promising alternative to the traditional GP-led model for MSK conditions, there is still a strong need to demonstrate that these pathways provide improvements in outcomes such as health care utilization, and opioid prescription in high quality, pragmatic RCTs.

### Strengths and Limitations

The strengths of this secondary data analysis include the use of a large dataset from a pragmatic RCT, and multiple imputed data analysis to reduce potential bias associated with missing data. Additionally, only a small number of plausible treatment effect modifiers and outcomes were selected a priori, on the basis of relevant literature, to minimize the likelihood of multiple testing leading to spurious findings.^29^^,^^61^

This study has limitations which must be considered when interpreting results. Using RCT data to determine prognostic factors may result in a more selective group of participants that influences the relationship between prognostic factors and outcomes compared to prognostic research models using a prospective inception cohort design. However, the pragmatic and cluster design of the STEMS RCT, and given treatment was administered in “real-world” clinics reflective of actual care, are likely factors that negate many of the biases typically resulting from a highly selected sample of patients.^24^ This study is an explorative secondary analysis. Although subgroups were of a reasonable size and balance (ie, age group >64 years included 111 and 177 participants in treatment and control groups respectively), the STEMS trial was not originally powered for subgroup analysis, and hence the available database had reduced power to detect a significant interaction effect. In addition, the prognostic variables and strength of association identified through backward stepwise regression methods, must be interpreted with caution owing to potential limitations of this method such as coefficient inflation.^62^ Interpretation of health care utilization and opioid prescription outcomes should acknowledge their respective limitations. First, health care utilization was collected using self-reported data and is subject to recall bias, potentially underestimating the utilization of health care.^51^ Health care utilization was grouped together to reduce the risk of producing spurious findings using multiple outcomes with a small number of events. However, this approach is not able to distinguish potentially important differences in associated prognostic factors or subgroups. It also places equal weighting on investigations and procedures which may have important clinical differences with respect to cost, burden, and potential patient harm. Also, the data collected did not allow a distinction between appropriate and inappropriate health care utilization. However, it does capture prognostic factors associated with an escalation of care, an important first step in identifying those at risk of higher health care utilization. Second, data on prior opioid use/disuse, previously shown to be associated with prolonged opioid use, was not available for analysis.^25^ In addition, opioid prescription rates analyzed over 6 months at 4 clinic sites, in the current study, might not be reflective of prescribing patterns over a longer period, in different areas of the United Kingdom or other health care systems. However, the prescription rates observed in the current analyses for patients with MSK conditions in primary care are comparable to rates observed in a similar cohort of patients analyzed across 25 UK general practices over 12 months, providing confidence in the prescription rates observed.^63^ Lastly, the authors acknowledge that elements of primary care practice may have changed since the conduct of the STEMS trial in 2013. However, “natural” follow-up of GP clinics participating in the STEMS trial^64^ and more recent evaluation of models of care offering first-contact physical therapy in the United Kingdom^65^ report uncertainty of the impact on health care utilization and patient clinical outcomes, reflecting original findings of the STEMS study.

### Implications and Conclusion

This study suggests that pain self-efficacy and pain severity are prognostic factors for clinical and health care outcomes, and having low back pain is a prognostic factor for future physical health and opioid prescription, in adults with MSK conditions consulting in primary care. Additionally, there may be important differences in the prognostic factors that predict clinical outcomes like physical health compared to health care utilization and opioid prescription. Although not directly synonymous with low value care, health care utilization and opioid prescription outcomes can reflect patients with complex needs, poor treatment response from other treatments, or unnecessary treatment.^27^ Identification of prognostic characteristics associated with these outcomes is an important first step in understanding which patients need a more focused early management approach to reduce unnecessary downstream health care utilization. We did not find robust evidence that patient characteristics identified a priori, modify the effectiveness of the offer of direct-access physical therapy compared to GP-led care alone. However, the trends observed that those of an older age and with greater duration of symptoms seemed to benefit more from the offer of direct access to physical therapy may inform the prespecification of treatment effect modifier selection in future RCTs. Further studies are needed to validate these findings to determine which patients benefit most from direct-access physical therapy models of care.

## Supplementary Material

2023-0221_R2_Supplementary_Material_au_cjt2_pzae066
