# Supplementary material for: Prognostic Factors and Treatment Effect Modifiers for Physical Health, Opioid Prescription, and Health Care Utilization in Patients With Musculoskeletal Disorders in Primary Care: Exploratory Secondary Analysis of the STEMS Randomized Trial of Direct Access to Physical Therapist–Led Care
Source: Phys Ther. 2024 May 2;104(8):pzae066. doi: 10.1093/ptj/pzae066 (PMC11365697; doi:10.1093/ptj/pzae066)
Supplement: 2023-0221_R2_Supplementary_Material_au_cjt2_pzae066 [file 2023-0221_r2_supplementary_material_au_cjt2_pzae066.pdf]

Supplementary files:

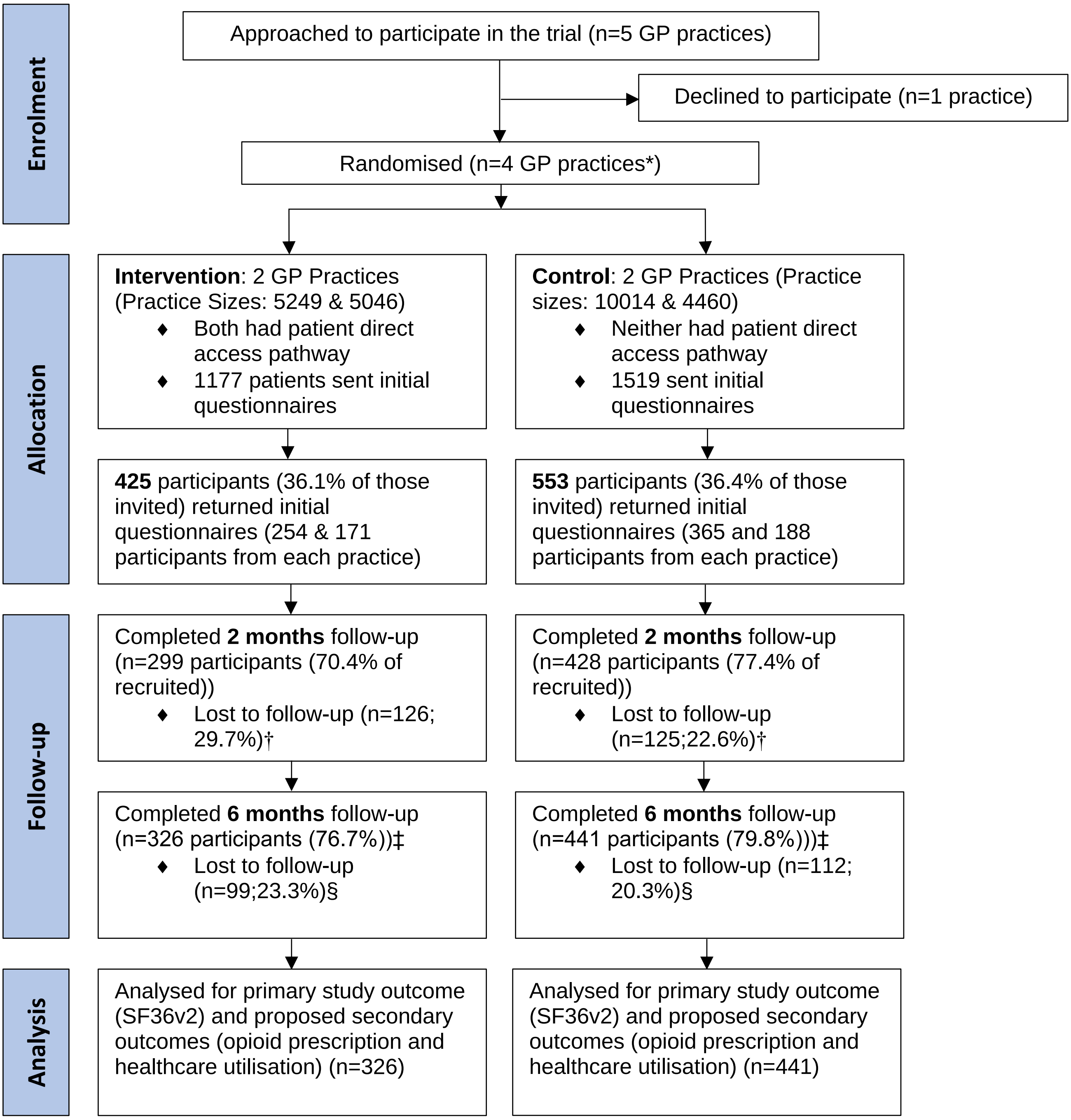

*All four randomised GP practices remained in the trial to the end
†Included 4 withdrawals, 2 from each arm
‡Includes minimum data collection on key outcomes
§Included 42 withdrawals, 21 from each arm
Reasons for withdrawal from 12-month follow-up data: subsequently ineligible (2), not wishing to continue (24), questionnaire burden (3), ill health (8), death (5), bereavement (2), moving house (7), not having any MSK problem (4), time commitment (3), unable to communicate in English (1), wrong person completing questionnaire (1), and moving to care home (1).

Supplementary Figure. STEMS pilot trial flow chart, modified with permission from Bishop et al.[28] GP = General Practitioner, MSK = Musculoskeletal.

Supplementary Table 1. Baseline characteristics/potential prognostic factors and measures

Potential prognostic factors	Measure/Outcome measure
Age	Self report (STEMS questionnaire)
Gender	Self report (STEMS questionnaire)
Ethnicity	Self report (STEMS questionnaire)
Employment	Self report, single question "Which of these best describes your current work situation…In paid work (**full-time** including self-employed), Unemployed/looking for work, Unable to work because of my pain problem, Unable to work because of other long term health problem, In full-time education or training, Other ?" (STEMS questionnaire)
Education level	Self report, single categorised question "What is your highest qualification?" (STEMS questionnaire)
Deprivation tertile	UK Index of Multiple Deprivation
Health literacy	Self report single question "Do you ever find health information or health related leaflets difficult to understand?" (STEMS questionnaire)
Physical health	SF36v2 Physical Component Summary
Mental health	SF36v2 Mental Component Summary
Bodily pain past week	Self report, single categorised question "How much **bodily** pain have you had during the **past week?"** rated none too severe (STEMS questionnaire), categorised into No/Mild, Moderate, Severe/Very Severe.
Episode duration	Self report, single categorised question "Have you had this current bout or episode of pain or related symptoms for…" (STEMS questionnaire), categorised into <6 weeks, 6-12 weeks, >12 weeks.
Widespread pain	Self report, coded body manikin completion using American College of Rheumatology definition of Widespread pain (STEMS questionnaire)
Comorbidities	Self report (STEMS questionnaire)
General Health	Self report, single categorical question "**In general**, would you say your health is Excellent, Very Good, Good, Fair, Poor" (STEMS questionnaire)
Pain self-efficacy	Pain Self Efficacy Questionnaire
MSK pain presentation	Self report, coded body manikin completion (STEMS questionnaire). Areas coded as low back pain labelled 'low back pain', all other conditions labelled as 'all other MSK presentations'.

Supplementary Table 2. Healthcare Utilisation by frequency at 6 months (complete cases)

Healthcare Utilisation frequency	Direct Access Arm (n=275)	Usual Care Arm (n=365)	Total (n=640)
0	175	227	402
1	73	100	173
2	24	26	50
3	1	10	11
4	2	2	4

Supplementary Table 3. Outcomes for healthcare utilisation by procedure at 6 months (complete cases)

Investigation/procedure type	Direct Access Arm (n=132)	Usual Care Arm (n=190)	Total (n=322)

MRI	33	35	68
X-ray	63	105	168
CT scan	6	10	16
Injection	18	22	40
Ultrasound scan	4	10	14
Surgery	8	8	16

Supplementary Table 4. Opioid prescription at 6 months (complete cases)

**Opioid prescription**	**Direct Access Arm (n=326)**	**Usual Care Arm (n=441)**	**Total (n=767)**
Yes	152	212	364
No	174	229	403

Supplementary Table 5. Multivariable linear regression model showing baseline prognostic factors associated with physical health (SF36v2 PCS) at 6 months (after controlling for intervention arm)

	**Multiple imputation**		**Complete Case Analyses**	
**Variable**	**b (95%CI)**	**p-value**	**b (95%CI)**	**p-value**
**Age** <45 years (ref)		p<0.001	**N/A**	**N/A**
45-64 years	-2.04 (-3.71, -0.37)			
Age >64 years	-3.59 (-25.38, -1.80)			
**Pain duration** pain <6 weeks (ref)		p<0.001		p=0.008
6-12 weeks	-1.40 (-3.52, 0.72)		-2.42 (-4.94, 0.10)	
>12 weeks	-3.16 (-4.61, -1.71)		-2.98 (-4.70, -1.27)	
**Bodily pain** no/mild (ref)		p<0.001		p<0.001
Moderate	-2.55 (-4.32, -0.78)		-2.92 (-5.12, -0.72)	
Severe-Very Severe	-4.49 (-6.54, -2.44)		-5.17 (-7.75, -2.59)	
**General health** Excellent (ref)		p<0.001		p<0.001
Very good	-2.61 (-5.05, -0.16)		-0.57 (-3.68, 2.53)	
Good	-5.81 (-8.23, -3.40)		-3.87 (-6.94, -0.82)	
Fair	-9.64 (-12.28, -7.00)		-7.76 (-11.11, -4.41)	
Poor	-11.40 (-14.74, -8.07)		-8.82 (-13.00, -4.65)	
**MSK pain presentation** all other (ref)				
Low back pain	-1.62 (-3.05, -0.19)	p=0.026	-2.94 (-4.51, -1.39)	p<0.001
**Comorbidities** 0-1 conditions (ref)				
2+ conditions	-2.30 (-3.62, -0.99)	p=0.001	-2.27 (-3.86, -0.69)	p=0.005
**Widespread pain**				
Yes	-1.60 (-3.07, -0.13)	p=0.033	**N/A**	**N/A**
**Pain self-efficacy (PSEQ)**	0.23 (0.17, 0.29)	p<0.001	0.23 (0.16, 0.30)	p<0.001
**Mental health (SF36v2 MCS)**	-0.07 (-0.13, -0.01)	p=0.019	-0.10 (-0.17, -0.03)	p=0.004
**Adjusted R^2**	0.52		0.52	

Table Legend
b= regression coefficient
CI= Confidence Interval
N/A= Not Applicable
Widespread pain: Based on the American College of Rheumatology's definition[66]

Supplementary Table 6. Variables with most missing data as a percentage of total data collected at 6 months

Variable	Missing (% of total)
Pain duration	42 (5.5%)
Level of Education	102 (13.3%)
Ethnicity	17 (2.2%
PCS	16 (2.1%)
PCS_6mth	29 (3.8%)
MCS	16 (2.1%)
MCS_6mth	29 (3.8%)
PSEQ	68 (8.9%)
Work	176 (22.9%)
Health Utilisation	127 (16.6%)

Supplementary Table 7. Distribution of the three outcomes for missing and complete observations

	Opioid use (%)		
**Variable**	**Missing**	**Complete**	**p-value**
Level of Education	59.8	51.4	p=0.115
Pain duration	50	52.7	p=0.734
work	60.7	50.8	P=0.036
PSEQ	58.8	51.9	P=0.277
	PCS, Mean (SD)		
Level of Education	37.0 (11.7)	38.5 (11.2)	p=0.219
Pain duration	39.4 (10.8)	38.2 (11.3)	p=0.521
work	37.3 (11.6)	38.5 (11.2)	p=0.254
PSEQ	37.0 (11)	38.4 (11.27)	P=0.337
	Healthcare Utilisation (yes %)		
Level of Education	41.0	36.6	p=0.445
Pain duration	20.6	38.1	p=0.040
work	34.2	37.4	p=0.695
PSEQ	30.4	37.8	p=0.268

Supplementary Table 8. Missing data patterns for key variables with the greatest proportion of incomplete observations

Variable	Health Utilisation	Level of Education	Paid work	PSEQ	Pain duration	PCS score, 6months
**Frequency of pattern**						
467	+	+	+	+	+	+
73	-	+	-	+	+	+
62	+	-	+	+	+	+
25	+	+	-	+	+	+
22	+	+	+	-	-	+
20	-	+	+	+	+	+
19	+	+	+	-	+	+
13	+	+	+	+	+	-
12	-	-	-	+	+	+
5	+	-	-	+	+	+

Key: + indicates observation was present – indicates observation was absent

Missingness: Missing data was investigated to determine the appropriateness of multiple imputation.[35–38] Firstly, the distribution of key variables between patients with complete and incomplete observations was analysed. There were differences in several variables including level of education, age, paid employment and PSEQ (supplementary Table 2). There was no clear pattern of 'missingness' between observations (supplementary Table 3). T-tests for the continuous outcome of physical health (PCS) and chi-squared tests for outcomes opioid use or healthcare utilisation (analysed as a binary outcome 'Yes' or 'No'), were used to compare the distribution of key outcome variables between complete and incomplete cases and provide information around the nature of missing data. There was evidence of a differences between those with incomplete and complete data for the variable *pain duration* and the outcome healthcare utilisation (p=0.04), and *paid employment* and the outcome opioid prescription(p=0.036). In addition, relationships between incomplete variables and potential predictor variables were examined using t-tests or chi-squared tests. There was strong evidence (p=0.002) of a difference in the level of deprivation for those with complete or incomplete observations for the variable level of education. These findings helped justify the Missing At Random (MAR) assumption used for multiple imputation in this secondary analysis.

Multiple imputation:
All variables used in analysis modelling, as well as predictive variables (deprivation level) were imputed using a multiple imputation by chained equations approach (MICE).[36]
A total of 40 datasets were imputed based on an established 'rule of thumb'[37,67] that suggests imputed sets should be at least equivalent to the total percentage of missing data. Continuous variables (PSEQ, physical component score, mental component score, and physical component score at 6 months) were imputed using linear regression. Although PSEQ was non-normally distributed it was imputed without transformation as only means and variance were important for subsequent regression analyses and this method has been reported to introduce limited bias.[68] A logistic regression was used to impute binary variables (work status, health literacy) and an ordered logistic regression model was used for ordered categorical variables (global assessment of change, general health at baseline, level of education, bodily pain at baseline and pain duration). A Poisson regression was used for healthcare utilisation imputation. Complete variables included in the imputation modelling were opioid prescription, widespread pain, sex, deprivation, area of pain, comorbidities, age, and treatment arm. A passive approach was used for interaction analyses which has been shown to be comparable to active imputation.[69] All variables were imputed together. A total of 40 data sets were imputed based on a 'rule of thumb' established in literature[37,67] that suggests imputed sets should be at least equivalent to the total percentage of missing data.

Imputation model checking:
The multiple imputation model was checked by firstly creating histograms and Kernel-density plots of the continuous variables in the imputed data sets and checking that values were plausible and, although variable, matched the distribution of the observed data set. PSEQ data had imputed observations outside the expected scale range, but mean and standard deviation of the imputed data sets were acceptable. Given the prior knowledge of its under-dispersed distribution, this was somewhat expected and not corrected for as

skewed variables imputed with a conditional normal distribution are known to produce acceptable estimates for means, variances and regressions which is the purpose of these analyses.[68] Categorical variables were tabulated to compare proportions against observed data. Kolmogorov–Smirnov tests were carried out on continuous data to compare the distribution of observed and imputed data sets. Abayomi et al suggested any variable with a significant test ($p<0.05$) is of potential concern.[70] No continuous variables in the model had a Kolmogorov–Smirnov test exceeding this value.

<u>VIF results:</u>
After computing the VIF, bodily pain at baseline had a VIF >10. Further exploration revealed PCS at baseline impacted the coefficients of multiple variables, inconsistent with the model, and removing this variable resulted in all remaining variables with a VIF <10.

Works Cited:

28. Bishop A, Ogollah RO, Jowett S, et al. STEMS pilot trial: A pilot cluster randomised controlled trial to investigate the addition of patient direct access to physiotherapy to usual GP-led primary care for adults with musculoskeletal pain. *BMJ Open*. 2017;7(3):1-11. doi:10.1136/bmjopen-2016-012987

35. Sterne JAC, White IR, Carlin JB, et al. Multiple imputation for missing data in epidemiological and clinical research: Potential and pitfalls. *BMJ*. 2009;339(7713):157-160. doi:10.1136/bmj.b2393

36. Harel O, Mitchell EM, Perkins NJ, et al. Multiple Imputation for Incomplete Data in Epidemiologic Studies. *Am J Epidemiol*. 2018;187(3):576-584. doi:10.1093/aje/kwx349

37. White IR, Royston P, Wood AM. Multiple imputation using chained equations: Issues and guidance for practice. *Stat Med*. 2011;30(4):377-399. doi:10.1002/sim.4067

38. Jakobsen JC, Gluud C, Wetterslev J, Winkel P. When and how should multiple imputation be used for handling missing data in randomised clinical trials - A practical guide with flowcharts. *BMC Med Res Methodol*. 2017;17(1):1-10. doi:10.1186/s12874-017-0442-1

66. Hill JC, Kang S, Benedetto E, et al. Development and initial cohort validation of the Arthritis Research UK Musculoskeletal Health Questionnaire (MSK-HQ) for use across musculoskeletal care pathways. *BMJ Open*. 2016;6(8):1-10. doi:10.1136/bmjopen-2016-012331

67. Bodner TE. What improves with increased missing data imputations? *Struct Equ Model*. 2008;15(4):651-675. doi:10.1080/10705510802339072

68. von Hippel PT. Should a Normal Imputation Model be Modified to Impute Skewed Variables? *Sociol Methods Res*. 2013;42(1):105-138. doi:10.1177/0049124112464866

69. Mitani AA, Kurian AW, Das AK, Desai M. Navigating choices when applying multiple imputation in the presence of multi-level categorical interaction effects. *Stat Methodol*. 2015;27:82-99. doi:10.1016/j.stamet.2015.06.001

70. Abayomi K, Gelman A, Levy M. Diagnostics for multivariate imputations. *J R Stat Soc Ser C Appl Stat*. 2008;57(3):273-291. doi:10.1111/j.1467-9876.2007.00613.x
